# Psychology of Fragrance Use: Perception of Individual Odor and Perfume Blends Reveals a Mechanism for Idiosyncratic Effects on Fragrance Choice

**DOI:** 10.1371/journal.pone.0033810

**Published:** 2012-03-28

**Authors:** Pavlína Lenochová, Pavla Vohnoutová, S. Craig Roberts, Elisabeth Oberzaucher, Karl Grammer, Jan Havlíček

**Affiliations:** 1 Department of Anthropology, Charles University, Prague, Czech Republic; 2 School of Natural Sciences, University of Stirling, Stirling, Scotland, United Kingdom; 3 Department of Anthropology, University Vienna, Vienna, Austria; Duke University, United States of America

## Abstract

Cross-culturally, fragrances are used to modulate body odor, but the psychology of fragrance choice has been largely overlooked. The prevalent view is that fragrances mask an individual's body odor and improve its pleasantness. In two experiments, we found positive effects of perfume on body odor perception. Importantly, however, this was modulated by significant interactions with individual odor donors. Fragrances thus appear to interact with body odor, creating an individually-specific odor mixture. In a third experiment, the odor mixture of an individual's body odor and their preferred perfume was perceived as more pleasant than a blend of the same body odor with a randomly-allocated perfume, even when there was no difference in pleasantness between the perfumes. This indicates that fragrance use extends beyond simple masking effects and that people choose perfumes that interact well with their own odor. Our results provide an explanation for the highly individual nature of perfume choice.

## Introduction

Odors are highly potent in affecting various domains of human psychological functioning, ranging from perception and mood to cognitive processes and behavior. Recent studies suggest that odors could be effective even at concentrations below conscious levels. For example, subthreshold ambient ‘sweet’ odors increase pain tolerance [1], while a common detergent perfume changes spontaneous cleaning behavior [2]. Further, results of recent studies suggest that odors can affect judgments of faces at both supra-threshold [3] and subliminal levels [4]. The last two studies are of particular importance as they indicate that odors can be involved in various social judgments, interactions and behavior. Indeed, the widespread use of fragrances in human societies may serve this same purpose.

Fragrance use is neither a recent phenomenon nor specific to western cultural settings, as historical records from ancient Egypt (and then later from ancient Greece and Rome) suggest that people commonly modified their body odor with a variety of odorous substances [5]. Numerous anthropological observations also point out that people of highly diverse cultures tend to manipulate their body odor in this way, suggesting that fragrance use is a near universal human phenomenon [6]. Furthermore, data on the still growing income of the cosmetics industry suggest that in modern times this is not an issue of marginal significance. For instance, and irrespective of various economic turnovers, estimated total sales in the fragrance and flavor industry rose from $12.9 billion in 1999 to $22 billion in 2010 [7].

Although fragrances appear to be used to rid the body of its underlying odor, growing evidence indicates that body odor plays a significant role in various social interactions and can carry important biological messages. To take just two examples: newborns are able to find their mothers' nipple by smell [8] while adults' judgments and decisions are influenced by the body odor of others who have experienced specific affective states (e.g. fear) [9]–[10]. However, it is thought that the principal context in which body odor influences social interactions is within romantic relationships and mate choice decisions in particular. Results of surveys in several western populations show that women report odor cues as most important in the context of partner choice [11]–[13]. Humans, similar to other species, are thought to partly base their choice on the genetic profile of the potential partner, exhibiting preferences for the odor of individuals who are dissimilar to themselves at genes in the Major Histocompatibility Complex (MHC) [14]. Products of these genes play a central role in immune system functioning and such disassortative preferences may therefore lead to offspring with more potent immune systems [15]–[16]. Furthermore, both men and women prefer the smell of individuals with lower fluctuating asymmetry, which is thought to be a marker of individual developmental stability [17]–[19]. It has also been found that women prefer the odor of men who are high in psychological dominance [20], that men prefer women's odor around ovulation compared to non-fertile cycle stages [21]–[22], and that odor samples collected at this time raise testosterone levels in men [23]. Lastly, some specific chemical constituents of human axillary sweat, notably androstadienone, have been repeatedly shown to affect heterosexual women's mood, physiology and social perception in both laboratory and semi-realistic settings [24]–[26].

All these findings point to the significance of body odors in social realms. However, as previously discussed, humans in various cultures engage in activities to modify or hide their body odor [6]. Why, then, do we live in a world of omnipresent personal fragrances? It has been proposed that using perfumes serves to indicate cleanliness, social status and personality [27]. Additionally, fragrances are frequently considered to enhance sexual attractiveness [28], and it has been found that they effectively modulate sexual arousal and mood response of females, particularly in the periovulatory phase of the cycle [29].

Moreover, perfume usage may also have an indirect impact on social perception through changes in the perfume wearer's self-perception and self-consciousness. For instance, Roberts et al. [30] asked their targets, half of whom were using a commercial deodorant (the other half used a placebo deodorant), to take a video recording while introducing themselves to an imagined person of the opposite-sex. An independent group of raters who saw the muted videos judged deodorant users as more attractive than the placebo group. Using a similar design, Higuchi et al. [31] also found changes in nonverbal behavior and increases in attributed confidence.

The above-mentioned studies suggest that enhanced attractiveness of perfume wearers is due to the masking effect of the perfumes. If this is the case, one would expect diminished variability between individuals in the pleasantness of their body odor when perfume is used (i.e. regression toward the mean). Milinski and Wedekind [32] proposed an alternative view, suggesting that people prefer to use perfume formulations that complement and enhance their own body odor, because they found a correlation between an individual's MHC profile (which affects body odor) and perfume ingredients preferred for oneself (but not for their partner). According to this view, the resulting odor retains characteristics of both perfume and body odor, with an emergent quality that is perceptually different from either constituent. If this is the case, then individual odor variability would be retained (or even enhanced) and perfume will affect the wearers' hedonics to a varying degree.

Here we set out to test between these two ideas. In two independent experiments, we compared hedonic ratings of perfumed and non-perfumed axillary samples obtained from the same group of donors. The studies were conducted in Vienna and Prague to examine cultural specificity of the studied phenomena. If perfumes mask body odor, we should find uniformly higher ratings of perfumed axillary samples and lower individual variability (i.e. no significant interaction). In the third experiment, we compared ratings of axillary samples collected while participants were wearing either their own preferred perfume or an assigned perfume. If the perfumes interact with the body odor in the manner proposed by Milinski & Wedekind [32], the ratings of axillary samples should be higher when wearing one's own perfume.

## Results

Initially, we tested the effect of perfume treatment on body odor perception using Wilcoxon matched-pairs tests. In Study 1, the perfumed samples were rated significantly more attractive (Z = 2.97; N = 29; p = 0.003) and pleasant (Z = 3.48; N = 29; p<0.001). Similarly, in Study 2, the perfumed samples were rated significantly higher on attractiveness (Z = 3.78; N = 20; p<0.001) and pleasantness (Z = 3.82; N = 20; p<0.001), and higher on intensity (Z = 2.72; N = 20; p = 0.006).

**Figure 1:**
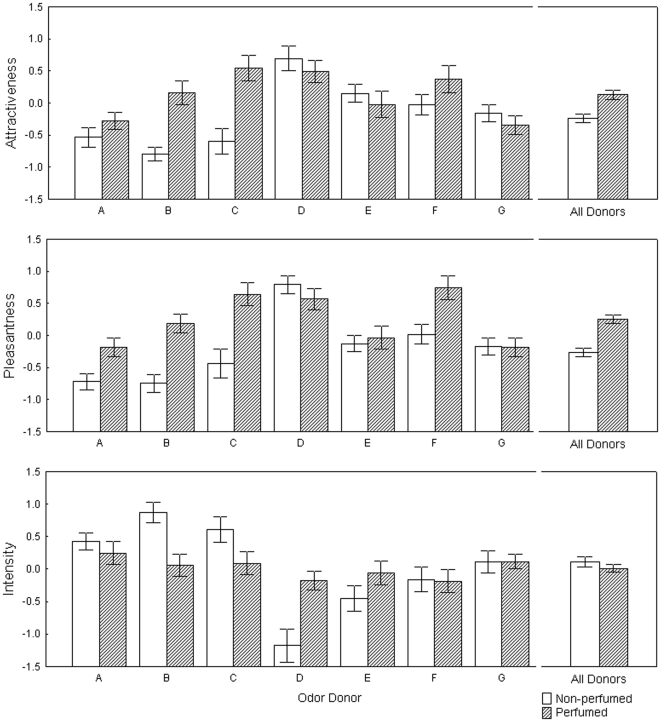
Ratings of perfumed and non-perfumed body odors in Study 1. Z-scored mean ratings (± SEM) of attractiveness, pleasantness and intensity in individual odor donors and for all donors together in non-perfume (empty bars) and perfume (shaded bars) conditions.

To test the interaction between individual body odor and perfume treatment on perception of the odor blends we performed repeated measures ANOVAs. In Study 1, we found a significant effect of individual donor on ratings of attractiveness, pleasantness and intensity (Table 1). The effect of perfume treatment was significant in ratings of attractiveness and pleasantness, but not in ratings of intensity. Compared to untreated ones (i.e. body odor only), perfumed armpit samples were rated as more pleasant and attractive. The interaction between individual donors and perfume treatment was highly significant for all rated variables (Figure 1) suggesting that the perfume affected individual donors differently. Finally, we did not find lower variance in the perfume condition in ratings of attractiveness (F_1,354_ = 2.06; p = 0.15) or pleasantness (F_1,354_ = 2.27; p = 0.13), lending support for the hypothesis of an interaction between axillary odor and the odor of the perfume, rather than to the hypothesis that perfumes simply mask human body odor. On the other hand, variance of ratings of intensity (F_1,354_ = 8.2; p = 0.004) was significantly lower in the perfume condition (Table 2).

**Table 1:** Results of ANOVA models for odor attractiveness, pleasantness and intensity in Study 1, 2 and 3.

	ID			Perfume			Interaction		
	F	p	η^2^	F	p	η^2^	F	p	η^2^
***Study 1***									
attractiveness	3.78	0.001	0.113	14.01	0.002	0.062	5.40	0.001	0.144
pleasantness	4.52	0.001	0.132	26.12	0.001	0.113	4.63	0.001	0.120
Intensity	4.06	0.001	0.120	0.27	NS	0.003	4.98	0.001	0.143
***Study 2***									
attractiveness	7.56	0.001	0.29	56.46	0.001	0.180	9.90	0.001	0.381
pleasantness	8.63	0.001	0.316	76.95	0.001	0.215	12.54	0.001	0.315
Intensity	5.45	0.001	0.226	19.40	0.001	0.080	6.02	0.001	0.224
***Study 3***									
attractiveness	9.75	0.001	0.313	10.94	0.001	0.031	9.84	0.001	0.306
pleasantness	9.90	0.001	0.317	13.23	0.001	0.036	10.75	0.001	0.322
Intensity	2.25	0.01	0.096	2.05	NS	0.008	3.21	0.001	0.130

Table shows values of test statistics (F), significance levels (p) and variance explained (η^2^) for factor donors identity (ID), odor condition (Perfume) and their interaction.

**Table 2:** Mean values, standard errors of the mean (SEM) and standard deviations (SD) for ratings of attractiveness, pleasantness and intensity of the axillary body odor (Non-perfumed) and perfume-body odor blend (Perfumed) in Study 1 and 2.

	Non-perfumed			Perfumed		
Study 1	Mean	SEM	SD	Mean	SEM	SD
attractiveness	2.48	0.11	1.57	3.09	0.12	1.74
pleasantness	2.47	0.11	1.57	3.23	0.12	1.72
Intensity	4.55	0.14	1.98	4.41	0.12	1.70

	Non-perfumed			Perfumed		
Study 2	Mean	SEM	SD	Mean	SEM	SD
attractiveness	3.71	0.12	1.55	4.76	0.13	1.72
pleasantness	3.92	0.13	1.68	5.01	0.13	1.67
Intensity	3.93	0.14	1.82	4.60	0.13	1.68

	Assigned perfume			Own perfume		
Study 3	Mean	SEM	SD	Mean	SEM	SD
attractiveness	3.33	0.11	1.68	3.73	0.13	1.98
pleasantness	3.44	0.11	1.71	3.88	0.13	2.02
Intensity	4.67	0.10	1.54	4.88	0.10	1.50

In Study 3 values are for perfume-body odor blend when using assigned and own perfume.

**Figure 2:**
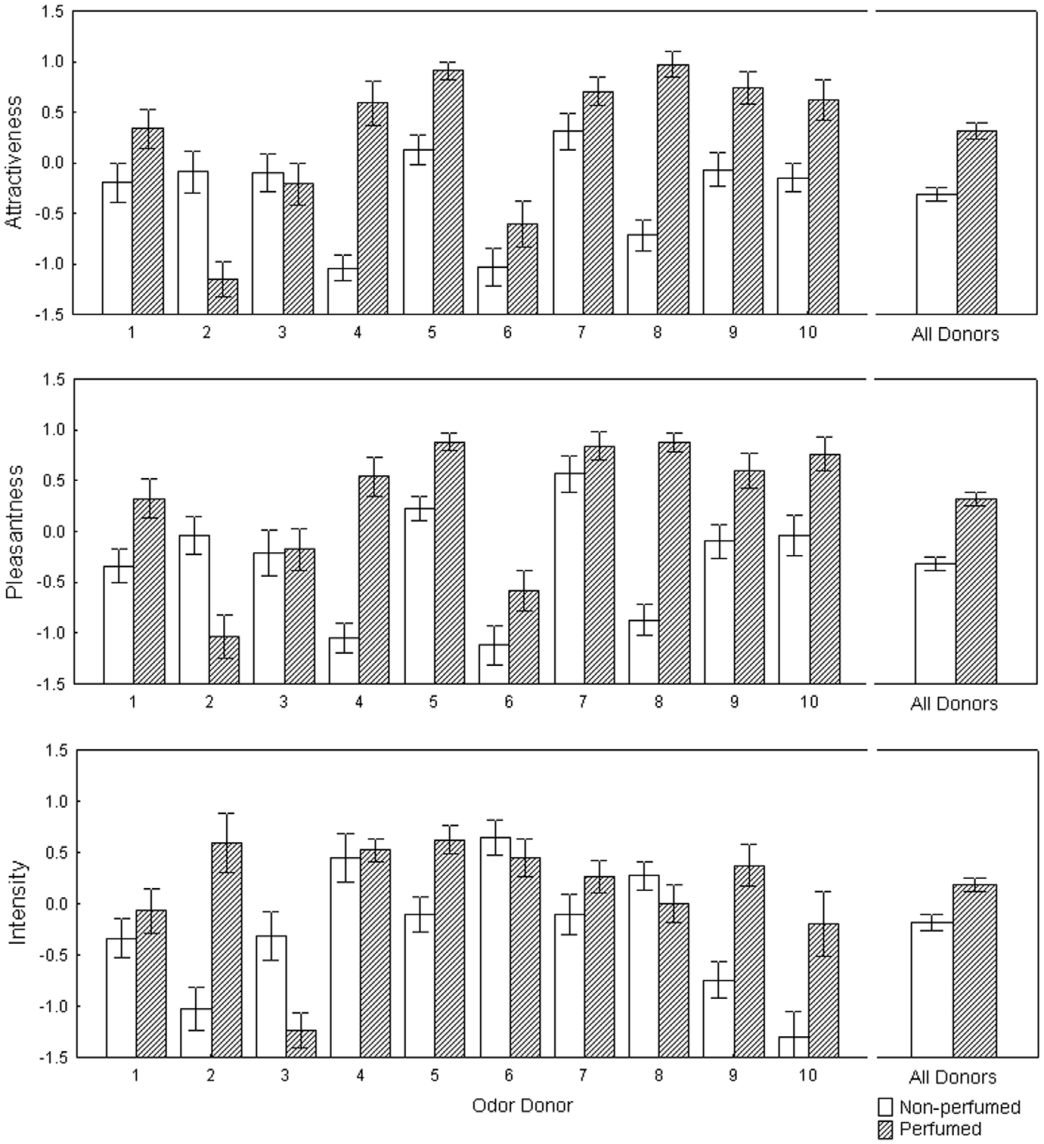
Ratings of perfumed and non-perfumed body odors in Study 2. Z-scored mean ratings (± SEM) of attractiveness, pleasantness and intensity in individual odor donors and for all donors together in non-perfume (empty bars) and perfume (shaded bars) conditions.

In Study 2, we similarly found a significant effect of individual donors on all dependent variables (i.e. attractiveness, pleasantness and intensity) (Table 1). The samples treated with perfume were rated as significantly more attractive, pleasant and intense than non-perfumed armpit samples. The individual donor and perfume treatment interaction was significant on all rated variables (Figure 2) and there was no significant difference in variance between perfume and control conditions in ratings of attractiveness (F_1,354_ = 0.99; p = 0.32), pleasantness (F_1,354_ = 0.67; p = 0.41) or intensity (F_1,354_ = 2.13; p = 0.15) (Table 2).

**Figure 3:**
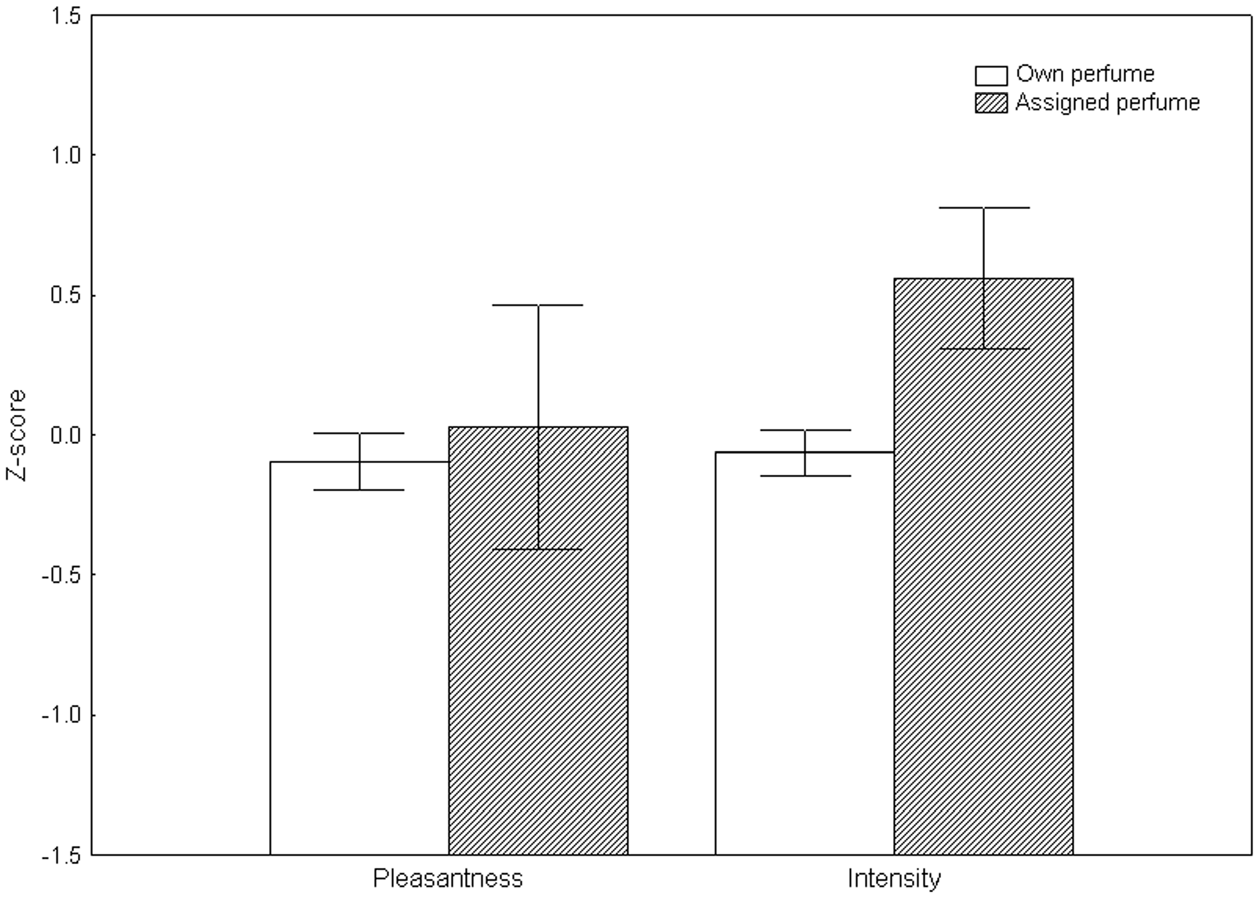
Ratings of own and assigned pure perfumes in Study 3. Z-scored mean ratings (± SEM) of pleasantness and intensity. Empty bars signify own and shaded bars assigned perfume.

In study 3, we first analyzed whether the pure perfumes (the assigned one and donors' own perfumes) were rated differently. Thus we compared the mean rating scores given to the donors' own perfumes with the mean scores of the assigned perfume, using Mann-Whitney U tests. We found a significantly higher intensity (Z = 2.03; p = 0.04) rating of the assigned perfume over the donors' own perfumes, but no significant difference in pleasantness (Z = 0.57; p = 0.57) (Figure 3).

**Figure 4:**
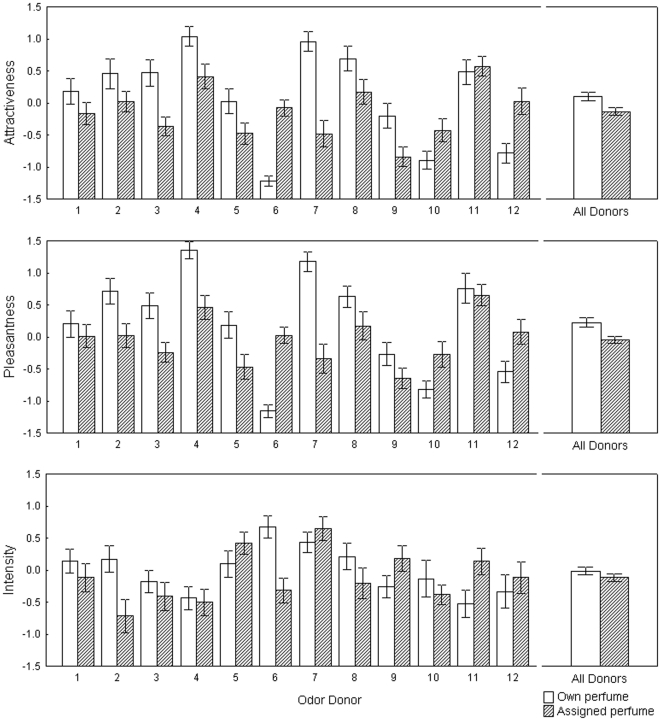
Ratings of own and assigned perfume-body odor blends in Study 3. Z-scored mean ratings (± SEM) of attractiveness, pleasantness and intensity of perfume-body odor blends in individual odor donors and for all donors together. Empty bars signify own and shaded bars assigned perfume-body odor blends.

Subsequently, we analyzed ratings of axillary odors when treated by the participant's own or the assigned perfume, using Wilcoxon matched-pairs tests. The odor blends with the participants' own perfume were rated significantly more attractive (Z = 2.37; N = 21; p = 0.02) and pleasant (Z = 2.48; N = 21; p = 0.01) than blends with the assigned perfume, but there was no significant difference in intensity.To test for interactions between individual body odor and perfume treatment, we used repeated measures ANOVA. In all rated variables we found a significant effect of individual odor donor. The axillary samples mixed with the perfume of participants' own choice were judged as significantly more attractive and pleasant (Table 2; Figure 4), but there was no significant difference in ratings of intensity (Figure 4). Further, similar to our previous studies, we found a significant effect of interaction between individual odor donor and perfume condition in all rated variables (Table 1).

## Discussion

In the first two experiments we found a positive effect of perfume on the perception of axillary samples, compared to the non-perfumed samples obtained from the same individuals. This is not suprising, as general attitudes toward untreated body odors is rather negative within European cultural settings [33]. However, this general effect of perfume usage was modified by the interaction with the target individual. Moreover, inspection of effect sizes, assessed by eta square (Table 1), shows that the effect of the perfume treatment was consistently weaker (e.g. for pleasantness 0.113, 0.215 and 0.036 in Study 1, 2 and 3 respectively) compared with the effect of the interaction (e.g. for pleasantness 0.120, 0.315 and 0.322 in Study 1, 2 and 3 respectively). This suggests that the impact of perfume varies among individuals, according to some aspect of the quality of body odor. Similar results of both studies thus lend support to Milinski & Wedekind's [32] notion of an interactive nature between perfumes and body odor rather than a simple masking effect. Although there is a myriad of different fragrances available to choose from, which might call into question the generalisability of our findings, the fact that our three studies used different perfumes and participants from two different countries (Austria and Czech Republic), with convergent results, suggests one underlying pattern rather than many fragrance-specific effects. This is in spite of the fact that cultural standards regarding use of personal care products, and perhaps also their perception, may be different in the two neighbouring countries, as a consequence of recent history. For instance, marketing, advertising and availability of various consumer goods, including fragrances, were relatively restricted in the Czech Republic until 1989. Although grooming habits have since changed dramatically, available data (e.g. perception of body odors) suggest that some specificities remain [11].

Although the main focus of our study (i.e. effect of the interaction) was consistent across the studies, we also found some discrepancies. The main difference between results of Study 1 and 2 was that intensity of perfume-body odor blends, compared to body odors alone, were rated significantly higher in Study 2 but not in Study 1. This could be due to specific perceptual properties of the perfumes used (‘B Men’- in Study 1 and ‘Hoggar’ in Study 2). However, this could also be attributed to the lower number of odor donors (and consequently, power) of Study 1. Thus, future studies should use several perfumes of varying perceived intensity to control for this effect. The results of the current study are restricted to the effect of perfume usage in male wearers. In theory, we might expect a similar pattern in women as well; however, as female axillary odors are weaker on average than those of men [34], [39], it is also plausible that the perfume would overpower the body odor. Future studies should address this question empirically.

Increases in positive attributions towards perfume wearers have been reported in several previous studies. Using the T-shirt method, Schleidt [34] showed that ratings of odor pleasantness were higher when participants used cosmetic products. Further, the overall effect of the perfume could be modified by other available cues and by situational context. For instance, Baron [35] found that formally dressed interviewers with perfume were judged as less attractive than those without perfume, but the opposite result was found for informally dressed interviewers. Despite all this, whether perfume usage has differential impact on relatively pleasant or unpleasant body odors (as judged by a panel of raters) remains unknown. Such an investigation would help to distinguish between the possibilities that our reported effects are due to individual-specific enhancement or individual-specific masking of body odor. It is a well-known phenomenon that mixtures of volatile chemicals have emergent perceptory qualities and that humans, including trained experts, perform rather badly in discrimination of individual components within the mixture [36]–[37]. However, while this mechanism may apply to odor discrimination, it may not be generalized to other cognitive processes such as hedonic perception. Our results suggest that, in terms of hedonic perception, the axillary odor and fragrance blend have emerging qualities while also retaining some of the qualities of its components. The exact mechanism of the interaction is not known, but there is evidence that volatile compounds in perfumes show different patterns of evaporation from human skin compared to an inert surface [38]. This could be due to body temperature, skin structure or presence of lipid particles, each of which can change temporal evaporation patterns of individual chemicals and thus also its perceptual quality.

Previous support for the masking hypothesis comes mainly from the finding that perfume use obscured correct gender attribution based on body odor [34]. This could be caused by the fact that gender of the body odor donor is usually attributed according to intensity of the sample rather than to any specific qualities [39]. However, Schleidt [34] found no significant reduction in terms of the individual odor identification, a result which is consistent with our findings.

The results of Study 3 suggest that people choose fragrances to complement their own odor, as the body odors blended with perfume of the participants' own choice were rated higher in pleasantness and attractiveness than when blended with the assigned perfume. Hedonic ratings of odors are usually interrelated [21], [39], thus one can argue that this could be due to higher ratings of intensity of the assigned perfume when rated alone. However, we think this is unlikely as we found no differences in the intensity of perfume and body odor blends. Further, and more importantly, when perfumes were rated alone, we found no significant differences in their hedonic quality. Thus the effect cannot be attributed to generally lower pleasantness of the assigned perfume. We deliberately recruited only participants who had chosen their own perfume, rather than using one given to them. Anecdotally, there are common complaints among perfumery customers that perfumes given to them do not really suit them and that, when choosing a perfume, they must try it on their own skin. This is in agreement with Milinski & Wedekind's [32] pioneering study which found a correlation between MHC profile and perfume preferences, but only when perfume ingredients were rated for self and not for a partner. An implication of this is that preferences for specific, genetically-linked body odor qualities, which mirror those found in animals and may be seen as adaptive preferences to increase offspring viability, may not be disrupted by cultural practices such as fragrance use. Indeed, these cultural practices may be exercised in full accordance with the underlying communicatory significance of body odor, rather than against them.

Evolutionary theorists of culture have repeatedly pointed out that cultural practices should be included into, and may significantly modify the outcome of, evolutionary models of human behavior [40]–[41]. In general, we concur with this view and we further suggest that the perfume-body odor complex may provide an insightful model into interactions between cultural and biological evolution. More specifically, various cultures prefer different substances suitable for fragrancing (e.g. [42]–[43]), based on local values and beliefs and on their local availability. However, fragrance use within individual communities is characterised by a high diversity of preferences. Our results indicate that people select specific perfumes that suit their individual body odor and they thus provide an insight into the highly individual nature of perfume choice. Furthermore, as particular fragrances appear to suit some individuals within the population more than others, patterns of individualised fragrance choice may create specific selective forces on body odor and fragrance use through differential patterns of mate choice. Consequently, and over generations, fragrance-related cultural practices may contribute to changes in genotype (and also phenotype) frequencies.

## Materials and Methods

### Study 1 and 2

#### Odor Donors

Seven men, University of Vienna students, aged 23–32 years (mean 25.9 years) with body weight 60–87 kg (mean 71.3 kg) and height 165–202 cm (mean 180.9 cm), participated as the body odor donors in Study 1. Ten male students of Charles University in Prague, aged 21–35 (mean 25.1 years) with body weight 67–90 kg (mean 76 kg) and height 170–197 cm (mean 180.3 cm) participated as odor donors in Study 2. None smoked, reported any serious disease, or shaved their armpits.

#### Raters

The odor samples in Study 1 were judged by 29 female students of Vienna University, aged 18–32 (mean 23.5 years). Fourteen used hormonal contraception. Cycle length reported by non-users varied between 26–31 days. The raters were also asked about the date of onset of their last menstrual bleeding (i.e. cycle day 1). Women in days 9–15 on the testing day were judged to be in the fertile phase and the others to be in the non-fertile phases of the cycle. Only three raters were in the fertile phase of their cycle, therefore we did not further test for the possible effect variation across the menstrual cycle.

Twenty female raters (Charles University students), aged 21–28 (mean 24 years) took part in Study 2. Here, to avoid the potential effect of fluctuations in olfactory function during the natural menstrual cycle, all were users of hormonal contraception [44].

The participants were contacted via posters, handouts given in lectures, advertisement on the University webpages, or personally by the first author. We recruited only participants without known smell damage or disorders and we further checked by a questionnaire that they did not suffer from any smell-related difficulty. However, we did not test for olfactory sensitivity, to reduce the burden on participants. All were given a 150 g chocolate bar and a perfume tester (Vienna) or cosmetics (e.g. shower gel or perfume) (Prague) in return for participation and gave oral informed consent (we did not ask for written consent as the nature of the study is non-invasive and participants' data were treated anonymously). Completed questionnaires were considered as a documentation of the oral consent. All three studies were carried out according to the Declaration of Helsinki and were approved (including oral consent) by the Institutional Review Board on Human Subjects of the Faculty of Science, Charles University in Prague. At the time of the study, no formal IRB for research involving human participants had yet been established at the University of Vienna's Faculty of Life Sciences.

#### Odor Sampling Procedure

Axillary odors were collected on cotton pads using the following procedure. The odor donors received a pack of experimental material (a white cotton T-shirt, a bar of non-perfumed soap, two cotton pads, a plaster and 2 zip-lock plastic bags). The cotton pads served as a medium for body odor collection (Study 1: 100% cotton pad, 10.5×6 cm, packed in aluminum foil with an oblong plaster (11×15 cm) attached to it; Study 2: 100% cotton, elliptical in shape, approximately 7 cm at their longest axis, attached by 3 M Micropore surgical tape). The donors were asked to follow the experimental schedule, including dietary and behavioral restrictions, on the day prior to sampling and on the sampling day. They were instructed to refrain from 1) using perfumes, deodorants, antiperspirants, aftershaves, and shower gels, 2) eating meals containing garlic, onion, chilli, pepper, vinegar, blue cheese, cabbage, radish, fermented milk products and marinated fish, 3) drinking alcoholic beverages or using other drugs, and 4) smoking. Additionally, they were asked to avoid exaggerated physical activities, sexual intercourse, and sleeping in the same bed with their partner or pet. All the necessary instructions were sent via e-mail several days before the experiment; if needed, ambiguities were discussed individually.

On the night before sampling the donors were instructed to use a non-perfumed soap (Sara Lee Household & Body Care, Stockholm, Sweden) and to wear a new white 100% cotton T-shirt, previously washed twice without washing powder, as the first layer of their clothing for the night and the following day to avoid odor contamination (e.g. other clothes, environment).

On the next day, the donors washed both armpits with the non-perfumed soap. Then donors in Study 1 applied 2 sprays of a perfume (‘B Men’ by Thierry Mugler) onto one, randomly assigned, armpit (the amount of perfume was determined on the basis of a pilot survey about common usage of perfume among the University students, N = 26), the other armpit was left untreated, serving as a non-perfumed control. The odor donors in Study 2 applied fragrance in the form of a wet-perfumed-tissue (‘Hoggar’ by Yves Rocher) by drawing the tissue three times over the armpit skin. Subsequently, all donors fixed the cotton pads into both armpits using the supplied materials and wore them for 24 hours (Study 1: starting at midnight; Study 2: at 7 am). On the next day, the donors returned the samples to the laboratory, where they were prepared for the rating session. The donors' conformity with the instructions was checked by a questionnaire. It showed no serious violations.

#### Odor Rating Procedure

The rating session started approximately an hour after the collection and continued for several hours (Study 1: 1 pm to 8:30 pm; Study 2: 9 am to 6 pm). All fresh samples were enclosed in clean 200 ml lidded plastic sniffing bottles (Study 1) or in 500 ml lidded glass opaque jars (Study 2) and code-labelled. In both cases, the ratings took place in a quiet, ventilated room. The samples were randomly split into two sets. After sniffing one set, the raters were recommended to have a 10-minute break to avoid possible odor habituation. During the break, the women were asked to complete an additional questionnaire. Order of sets, and order of stimuli within a set, was randomized for each rater.

The stimuli were assessed on 7-point scales for their 1) pleasantness, 2) attractiveness, and 3) intensity, anchored by verbal descriptors (e.g. very un/pleasant). As in previous studies (e.g. [19]–[21]), the ratings were written down immediately after sniffing each stimulus, but the time spent by sniffing and the intervals between individual samples were not restricted in order to make the procedure more convenient for the raters. Raters were instructed to select the expression “I cannot smell the sample”, instead of using the scales, if they found a sample too weak to detect; 18 of 140 rating pairs (i.e. perfumed and non-perfumed samples collected from the same individual) in Study 1 and 22 of 200 in Study 2 were excluded for this reason, leaving 128 and 178 pairs, respectively, to enter the subsequent analysis.

The studies slightly differed in several aspects of the odor data collection. These differences involved the timing of the odor collection, size of cotton pads and sniffing bottles. These differences mainly reflect different traditions in the two labs and to our knowledge, there are no relevant methodological studies testing their potentially confounding effects; for full discussion on this issue, see [45]–[46]. However, we can think of no way that this should introduce any systematic bias to the data that is relevant to the tested hypothesis.

#### Statistical Analysis

Dependent variables were assessed on 7-point scales and the study design was within-subject, therefore we used the Wilcoxon matched-pairs test for testing the effect of the treatment (perfumed vs non-perfumed) with mean values for each rater as the unit of the analysis. However, this method does not allow for testing of interactions (ID × perfume condition) which is crucial in our analysis. As ANOVA is relatively robust to deviations from normality [47], we used a repeated measures ANOVA with perfumed/non-perfumed condition as a repeated measure and individual odor donor as a between-subject factor, and rated characteristics (e.g. attractiveness) as dependent variables. To control for the potential effect of hormonal contraception use in Study 1, we included this variable in the analysis as another between-subject factor. However, we found no significant main effect of hormonal contraception use, its interaction with individual donor, or with perfume treatment on ratings attractiveness, pleasantness or intensity, so we excluded this variable from further analysis. Strength of the effect was assessed by eta-squared (η^2^) and homogeneity of variance by Levene's test. The statistical package Statistica 7.1 was used for all analyses.

### Study 3

#### Odor Donors

Twelve men, aged 21–28 years (mean 23 years), with body weight 65–90 kg (mean 75 kg) and height 173–188 cm (mean 182 cm), participated as the body odor donors. None reported any serious disease. All participants used a perfume that they had personally chosen (not a gift bought by someone else) and which they found pleasant. None of the donors used the same brand of perfume. All donors reported using perfumes on a regular basis. Regarding frequency of usage, seven donors (58.3%) stated using the chosen perfume at least once a day and the rest did not specify. They were reimbursed for their participation by 300 CZK (approx. 25 USD).

#### Raters

Samples of axillary odor with perfume were judged by 21 women, aged 17–37 (mean 23 years). All were using hormonal contraception. Raters were given a 150 g chocolate bar and a perfume tester after participation. In addition, pure perfume samples were assessed by 15 women – hormonal contraception users, aged 19–30 (mean 23 years). All participants were Charles University students, were recruited via email or personally by the second author, and signed informed consent.

#### Odor Sampling Procedure

Restrictions in diet, hygienic practices and activity were identical to Studies 1 and 2. Donors were asked to follow the instructions during the two days prior to, and on the day of sampling. At midnight they applied two sprays (as in Study 1) of their own perfume to one, randomly chosen, armpit, and the same amount of an assigned perfume (identical for all participants) to the other armpit. The assigned perfume was Balea Men Electric Blue and none of the participants used it as his own. Subsequently, they applied pads (see Study 2 for details) with surgical tape to their armpits and wore them for 12 hours (12 pm to 12 am).

#### Odor Rating Procedure

The rating session started within 2 h of samples collection (12 am) and finished approximately at 7:30 pm. It was conducted in a quiet, ventilated room. All 24 odor samples (2 from each donor) were encased in 500 ml lidded glass opaque jars and randomly split into three sets. The samples from each donor were presented in pairs and rated in the form of a forced choice test. Order of sets and order of stimuli within a set was randomized for each rater. All other details were identical to Studies 1 and 2.

For ratings of the pure perfume samples, we applied the same amount of perfume as in the previous part of the study onto cotton pads and encased them in zip-locked plastic bags. The order of samples was randomized and they were rated on a 7-point scale for pleasantness and intensity by a different group of female raters.

#### Statistical Analysis

Similarly to Study 1 and 2, we used Wilcoxon matched-pairs tests to test the effect of the treatment (own perfume vs assigned perfume) and repeated measures ANOVA to test for the interaction (ID × perfume condition). Further, we used Mann-Whitney U tests to test for differences between ratings of the pure perfume samples.
